# A New Transgenic Mouse Line for Imaging Mitochondrial Calcium Signals

**DOI:** 10.1093/function/zqab012

**Published:** 2021-02-25

**Authors:** Nelly Redolfi, Elisa Greotti, Giulia Zanetti, Tino Hochepied, Cristina Fasolato, Diana Pendin, Tullio Pozzan

**Affiliations:** 1 Department of Biomedical Sciences, University of Padua, Via U. Bassi 58/B, 35131 Padua, Italy; 2 Neuroscience Institute, Italian National Research Council (CNR), Via U. Bassi 58/B, 35131 Padua, Italy; 3 VIB Center for Inflammation Research, Ghent, Belgium; 4 Department of Biomedical Molecular Biology, Ghent University, Ghent, Belgium; 5 Veneto Institute of Molecular Medicine (VIMM), Via G. Orus 2, 35129 Padua, Italy

**Keywords:** Cameleon, calcium imaging, transgenic mouse, mitochondria, ROSA26, Cre/loxP, GECI, FRET, mt-Cam

## Abstract

Mitochondria play a key role in cellular calcium (Ca^2+^) homeostasis. Dysfunction in the organelle Ca^2+^ handling appears to be involved in several pathological conditions, ranging from neurodegenerative diseases, cardiac failure and malignant transformation. In the past years, several targeted green fluorescent protein (GFP)-based genetically encoded Ca^2+^ indicators (GECIs) have been developed to study Ca^2+^ dynamics inside mitochondria of living cells. Surprisingly, while there is a number of transgenic mice expressing different types of cytosolic GECIs, few examples are available expressing mitochondria-localized GECIs, and none of them exhibits adequate spatial resolution. Here we report the generation and characterization of a transgenic mouse line (hereafter called mt-Cam) for the controlled expression of a mitochondria-targeted, Förster resonance energy transfer (FRET)-based Cameleon, 4mtD3cpv. To achieve this goal, we engineered the mouse *ROSA26* genomic locus by inserting the optimized sequence of 4mtD3cpv, preceded by a *loxP*-STOP-*loxP* sequence. The probe can be readily expressed in a tissue-specific manner upon Cre recombinase-mediated excision, obtainable with a single cross. Upon ubiquitous Cre expression, the Cameleon is specifically localized in the mitochondrial matrix of cells in all the organs and tissues analyzed, from embryos to aged animals. Ca^2+^ imaging experiments performed *in vitro* and *ex vivo* in brain slices confirmed the functionality of the probe in isolated cells and live tissues. This new transgenic mouse line allows the study of mitochondrial Ca^2+^ dynamics in different tissues with no invasive intervention (such as viral infection or electroporation), potentially allowing simple calibration of the fluorescent signals in terms of mitochondrial Ca^2+^ concentration ([Ca^2+^]).

## Graphical abstract

**Figure:**
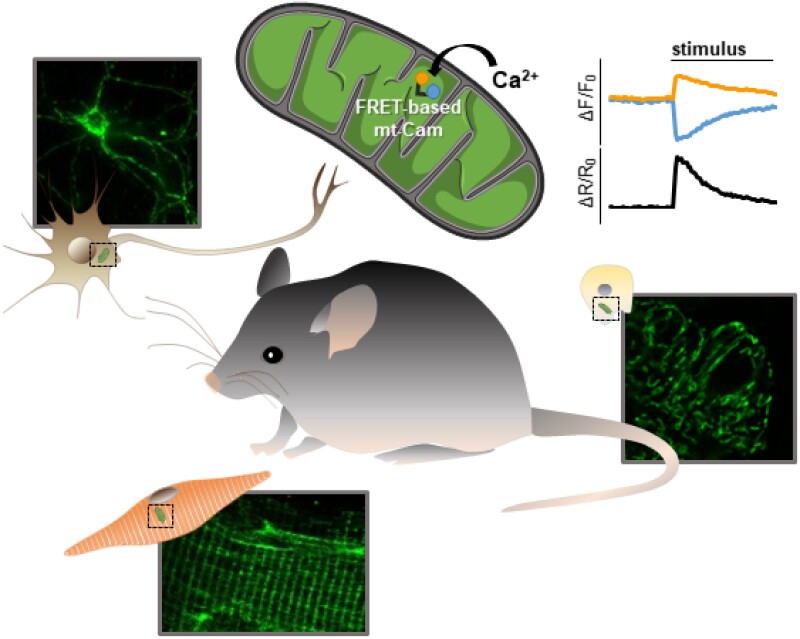

## Introduction

Precise regulation of cytosolic [Ca^2+^] in terms of amplitude, frequency, and spatiotemporal patterns contributes to fundamental processes such as synaptic transmission, exocytosis, muscle contraction, and cell death.^1–4^ These processes are finely orchestrated and even subtle changes give rise to a disequilibrium in the system and correlate with pathologies ranging from cardiovascular diseases to neurological disorders and carcinogenesis.^5–7^

Cytosolic [Ca^2+^] is not only controlled by the action of plasma membrane (PM) pumps, channels, and exchangers, but also by the presence of numerous Ca^2+^ buffers and by the activity of organelles, *i.e.* primarily, the endo/sarcoplasmic reticulum (ER/SR), the Golgi apparatus and mitochondria.^8–11^ Best known for their role in energy metabolism and cell death,^12^ mitochondria are nowadays well accepted as central players of the cellular Ca^2+^ handling toolkit.^8^^,^^13–18^ Beside contributing to shaping cytosolic Ca^2+^ signals, mitochondria rely on Ca^2+^ for their specific metabolic functions^19–21^ and dysfunctions of the mitochondrial Ca^2+^ handling machinery are linked to several pathological conditions.^22–29^

Advances in understanding of biological phenomena have almost invariably been dependent on improvements in the techniques to measure them and this is particularly true in the case of Ca^2+^ signaling.^30–32^ In particular, the introduction in the last decades of more and more sensitive and sophisticated Ca^2+^ indicators, both synthetic and genetically encoded, has been essential for unraveling the role of Ca^2+^ in cell pathophysiology.^32^^,^^33^ Mitochondria are a classical example of how technology and physiology are intimately linked. Until the 90s, the lack of methods to measure Ca^2+^ dynamics in the mitochondrial matrix led to the wrong conclusion that these organelles were not involved in cellular Ca^2+^ signaling. The development of new methods that allowed the monitoring of mitochondrial matrix Ca^2+^ in living cells completely changed this scenario. Today, a large number of methodologies to monitor mitochondrial Ca^2+^ in living cells is available, from chemical dyes to GECIs.^4^^,^^34–39^ Mitochondria-targeted GECIs, in particular, have extensively been used in cell lines where transfection is, in most cases, a simple and efficient method to express the sensors. More complex is the expression of GECIs in primary cultures, acute slices, and especially *in vivo*, where infection with viral vectors or electroporation protocols are the exclusive choice. We and others have exploited *in vivo* virus-mediated delivery or electroporation of mitochondria-targeted recombinant Ca^2+^ probes.^4^^,^^39–44^ Although of undeniable value, these approaches present important limitations, for example, invasive surgical delivery that is time-consuming and stressful for the experimental animal, limited spread of the virus in the region of interest, uneven expression in the target cell population, potential cytotoxicity, or high gene expression.^45^

Ideally, to overcome these issues, the use of transgenic mice expressing Ca^2+^ sensors in a controlled way appears the best solution and, indeed, several mouse lines expressing cytosolic GECIs have become available in the last years.^46–52^ Transgenic animals expressing GECIs offer a number of advantages over viral injection (or other techniques such as *in situ* electroporation and bulk injection), guaranteeing less invasive experimental procedures, consistent expression level among cells and during aging, full access to all body regions. Surprisingly, however, no such transgenic mouse lines are available yet for mitochondria. A couple of lines have been generated for the expression of mitochondria-targeted bioluminescence-based sensors (GFP-aequorin,^53^ c-Photina^54^) but the spatial resolution of these sensors is quite limited. Moreover, these probes are non-ratiometric and thus are sensitive to motion artifacts of the sample due to breathing, heartbeat, and blood flow or movements of the organelles themselves. Anecdotical information from different laboratories tells that, although mitochondria-targeted sensor sequences can be easily inserted in the mouse genome, the level of expression of the Ca^2+^ indicators are so low to be practically useless. The reason for these repetitive failures is presently unknown.

We here describe the generation and characterization of a transgenic mouse line expressing the mitochondria-targeted Cameleon Ca^2+^ sensor 4mtD3cpv^55–57^ under the control of Cre recombinase. Among GECIs, we focused on this sensor because, though not the most brilliant among the available mitochondrial Ca^2+^ sensors, it is based on FRET and accordingly: (1) it allows ratiometric measurement of signal dynamics and thus, unlike intensity-based indicators, permits easily to transform the fluorescence changes into absolute [Ca^2+^] values; (2) it guarantees that the output measurement is independent of movement artifacts, probe expression levels, focus, and intensity changes.^4^^,^^58^ Mitochondria, in particular, are highly dynamic organelles that undergo fission and fusion processes and move inside the cell. For these reasons, ratiometric biosensors are particularly recommended for qualitative and quantitative Ca^2+^ measurements in these organelles, ensuring independency from movements and shape changes. Cameleon 4mtD3cpv is characterized by a single affinity constant for Ca^2+^ (*K*_d_ ≈ 3.2 µM at pH 8) and a dynamic range in response to Ca^2+^ changes (*R*_max_/*R*_min_) calculated around 2.4 in living cells,^39^ features that make this probe suitable to measure mitochondrial Ca^2+^ changes in different organs and tissues.

To generate the transgenic mouse line mt-Cam, the Cameleon sequence, preceded by a *loxP*-STOP-*loxP* sequence, was inserted in the *ROSA26* genomic locus, well characterized for ubiquitous expression of transgenes at moderate levels without interrupting essential genes.^59^^,^^60^ The probe can be expressed in a tissue-specific, controlled manner upon Cre recombinase-mediated excision, easily obtainable with a single genetic cross. Following crossing with ubiquitous or tissue-specific Cre-expressing mice, positive offspring showed expression of the mitochondria-targeted probe in different cell types, from newborn to aged animals. Moreover, a series of classical Ca^2+^ imaging experiments in primary cultures and in acute brain slices are presented here, demonstrating the functionality of the probe and its potential applicability to other relevant experimental paradigms.

## Materials and Methods

### Construction of the Probe

To improve the transduction efficiency in mammalian cells, the mitochondria-targeted Cameleon sequence was optimized for mouse codon usage (GeneArt Gene Optimizer service from Life Technology). The codon-optimized version of 4mtD3cpv cDNA sequence, modified as previously reported,^39^ was obtained by chemical synthesis (GeneArt Gene Synthesis service from Life Technology). Briefly, a Kozak consensus sequence (GCCACC) was placed before the starting codon and the cDNA sequence encoding the 33 aminoacid-long N-terminal sequence derived from COX VIII was repeated 4 times to guarantee mitochondrial matrix import. The resulting nucleotide sequence (named mt-Cam) is available upon request.

### Gateway Vectors Construction and Gateway Reaction

MultiSite Gateway Cloning was used to create a conditional *ROSA26*-targeting vector. The vector consisted in a CAG promoter *floxed* βgeo-STOP cassette^59^ placed before mt-Cam coding sequence, inserted between a 5′ and a 3′ *ROSA26* locus homology arm in anti-sense orientation relative to the *ROSA26* sense promoter. The resulting pROSA26-mt-Cam vector is depicted in Supplementary Figure 1 and its construction was obtained through the recombination of pROSA26 destination and 3 Entry vectors, as follows.

The MultiSite Gateway-compatible destination vector pROSA26-DV2 (LMBP 6452) and the Entry vector attL4-pCAGG-loxP-bgeo-3xpA-loxP-attR1 (LMBP 6455) were previously described^59^ and are available from the BCCM/GeneCorner Plasmid collection, Department of Biomedical Molecular Biology, Ghent University, Belgium (https://www.genecorner.ugent.be/; bccm.genecorner@ugent.be).

Entry vector attR2-IRES-eGFP-luc+-pA-attL3 (LMBP 8200^61^) was modified in order to eliminate the sequence coding for GFP. Briefly, LMBP 8200 vector was cut with AleI-AgeI, gel purified and ligated after blunting ends with T4 DNA Polymerase.

The attL1–attL2 Entry vector was created by cloning the codon-optimized version of 4mtD3cpv cDNA sequence mt-Cam in pENTR1A (Thermo Fisher Scientific) using restriction enzymes KpnI-XhoI.

MultiSite Gateway LR reaction was performed using Gateway LR Clonase II Enzyme mix (Thermo Fisher Scientific) according to the suppliers’ instructions. Briefly, 50 ng of each of the 3 Entry vectors were first incubated with the LR Clonase II enzyme mix for 6 h at room temperature (RT), then 150 ng of pROSA26-DV2 Destination vector was added with additional Clonase II enzyme mix and incubated overnight (O/N) at RT. LR reaction was desalted using 0.025 µm membrane (Millipore) prior to electroporation (1.5 kV) in One Shot TOP10 electrocompetent bacteria (Thermo Fisher Scientific).

Bacteria were recovered in lysogeny broth LB medium without antibiotics at 28°C for 90 min shaking and plated on LB-AMP agar plates also grown at 28°C to avoid recombination. Correctly recombined clones were selected by restriction analysis with KpnI or XhoI, followed by sequencing.

### ES-cell Culture and Electroporation

G4 embryonic stem (ES) cells were cultured in standard ES-cell medium containing in 500 mL of KnockOut Dulbecco’s modified Eagle’s medium (DMEM) (Thermo Fisher Scientific): 90 mL fetal bovine serum (FBS) (HyClone), 6 mL 1× Nonessential amino acids (Thermo Fisher Scientific), 6 mL 1× l-Glutamine (Thermo Fisher Scientific), 6 mL of 0.07% (v/v) β-mercaptoethanol in Phosphate-Buffered Saline (PBS) (Thermo Fisher Scientific), and 2000 U·mL^−1^ leukaemia inhibitory factor (Protein Service Facility, VIB, Ugent). Culture dishes were kept at 37°C in a humidified atmosphere with 5% CO_2_. ES cells were subcultured every 2–4 days onto mitomycin-C inactivated feeder layers, and fed every day with fresh cell culture medium.

For electroporation, ES cells were harvested by trypsinization with 0.25% trypsin plus 1 mM Ethylenediaminetetraacetic acid (EDTA) (Thermo Fisher Scientific) and washed with PBS (Thermo Fisher Scientific). The cell suspension was diluted with PBS to a final concentration of 0.75 × 10^7^ cells·mL^−1^. 800 µL cell suspension (0.6 × 10^7^ cells) were mixed with 25 µg of linearized pROSA26-mt-Cam targeting vector in an electroporation cuvette (0.4 cm gap, Bio-Rad Laboratories). Electroporation was performed using a gene pulser II electroporator (Bio-Rad Laboratories), setting 200 V and 500 µF. After electroporation, the cells were seeded onto a 10 cm culture dish containing neomycin-resistant, mitomycin-C inactivated feeder cells. Twenty-four hours after electroporation, G418 (150 µg·mL^−1^) (Thermo Fisher Scientific) and 4 days after the electroporation, ganciclovir (2 µM) (Sigma-Aldrich) were applied to the ES cells. Nine days after the electroporation, surviving colonies were picked.

### PCR and Southern Blot Analysis

Polymerase chain reaction (PCR)-based screening of *ROSA26*-targeted ES cell clones was performed using mt-Cam specific primers (Primer F: 5′-TTCCTGTGTGAAAGGGCGAA-3′, Primer R: 5′-CAGAGAGCCTCGGCTAGGTA-3′) to generate a 1.3 kb PCR fragment. PCR positive clones were confirmed by Southern blotting for 5′-integration using a 5′-external probe (550 bp) after EcoRI-KpnI digestion of genomic DNA (4.2 kb fragment in correctly targeted allele, 11 kb fragment for the un-targeted wild-type allele). 3′-integration was confirmed using a 3′-external probe (800 bp) after EcoRI-KpnI digestion of genomic DNA (7.8 kb fragment for correctly targeted allele, 11 kb fragment for the un-targeted wild-type allele). Both the 5′- and 3′-external Southern probes were generated by Michael Taschner and Christine Hartmann (IMP, Vienna). An internal probe on mt-Cam (800 bp) was used to detect a single 4.9 kb fragment in mt-Cam *ROSA26*-targeted allele, upon BamHI digestion of genomic DNA. Southern blots were performed following standard procedures, using Digoxigenin (DIG)-High Prime DNA Labeling and Detection Kit (Roche, #11585614910) for random-primed labeling of DNA probes and chemiluminescent detection of the DIG-labeled hybrids.

### Blastocyst Injection

Blastocyst injection was carried out by injecting 15–25 correctly targeted ES cells into host blastocysts of C57BL/6J mice. After injection, blastocysts were re-implanted into 2.5-day pseudo-pregnant Swiss Webster females, previously mated to vasectomized males. Chimeric animals were mated to C57BL/6J mice to generate heterozygous germline offspring. Resulting offspring were genotyped to check for the presence of the targeted insertion.

### Mouse Lines

All animal procedures were performed following a protocol approved by the ethic committee of the University of Padua and the Ministry of Health (287/2015-PR). ROSA26-mt-Cam female mice were crossed with male mice expressing Cre recombinase under the control of a cytomegalovirus immediate early enhancer chicken β-actin hybrid (CAG) promoter (CAG-Cre mice^62^) or the tissues specific promoter of Choline Acetyltransferase (ChAT-IRES-Cre mice, stock #006410 from The Jackson Laboratory). The F1 generation was used for experiments.

### PCR Analysis on Genomic DNA

Genomic DNA extraction from mouse tissues was performed with KAPA Express Extract Kit (Kapa Biosystems) following the manufacturer’s instruction. PCR reactions were performed with the same kit following KAPA2G Robust PCR Protocol (Kapa Biosystems).

The following primers were used:

- to assess the presence of the Cameleon (Primer R: 5′-CTAGGTAGGGGATCGGGACT-3′, Primer F: 5′-GTGTCTCTCACTCGGAAGGAC-3′, resulting in a 1660 bp amplicon; Primer R: 5′-GAGAGTGAATTTTGGCCCTG-3′, Primer F 5′-ACTTCCTTTGTCCCAAATCTG-3′, resulting in a 930 bp amplicon).
- to assess the presence of the Cre (Primer R: 5′-TTACATTGGTCCAGCCACCAG-3′, Primer F: 5′-CACCAGCCAGCTATCAACTCG-3′, resulting in a 230 bp amplicon).
- to verify the excision of the βgeo-STOP cassette (Primer R: 5′-CAAAACAGGCGGCAGTAAG-3′, Primer F: 5′-ACGGAAGCAAAACACCAG-3′, resulting in a 900 bp amplicon).

### Quantitative PCR

Total RNA was extracted from 20 mg of each tissue using the NucleoSpin RNA purification kit (Macherey-Nagel, 740955.250) and quantified with NanoDrop 2000 (Thermo Fisher Scientific). For each sample, 1 μg of total RNA was reverse-transcribed into cDNA using the SuperScript II Reverse Transcriptase (Thermo Fisher Scientific, 18064071) following the manufacturer’s instructions. PCR amplification was performed in a CFX96 Touch Real-Time PCR Detection System (BioRad), using Applied Biosystems PowerUp SYBR Green Master Mix (Thermo Fisher Scientific, #A25777). The transcript of TOM20 was used as an internal control. The following primers were employed:

mt-Cam: 5′- TCAGGCACAACATCGAGGAC-3′ and 5′- GTGGTCCCTCTTCTCGTTGG-3′

TOM20: 5′- CTGACAAATGCCATTGCTGT-3′ and 5′-CACATCATCTTCAGCCAAGC-3′

Data are expressed as percentage change relative to the internal control transcript.

### Western Blot Analysis

Protein extraction from tissues was performed using GRS Full Sample Purification Kit (GRiSP) according to the manufacturer's instructions. The protein pellet was solubilized in 200 μL of RIPA Buffer (50 mM Tris, 150 mM NaCl, 1% Nonidet P-40, 0.5% deoxycolic acid, 0.1% sodium dodecyl sulfate (SDS), pH 7.5), supplemented with proteases and phosphatases inhibitor mixtures (Roche, #04693132001 and #04906837001) and 3 M urea, then sonicated for 30 min at 37°C. Insoluble particles were spun down at 10 000*g* for 5 min. Protein concentration was measured by bicinchoninic acid (BCA) assay (QuantumProtein assay, EuroClone). Western blot was performed following standard procedures. Briefly, 20 μg of protein were loaded per lane and separated by SDS-polyacrylamide gel electrophoresis (SDS–PAGE) (10% polyacrylamide gel), transferred into nitrocellulose membranes (GE Healthcare, #10600001) and probed using the following antibodies: rabbit anti-GFP, 1:1000, Cell Signaling #2956; mouse anti-HSP90, 1:1000, BD Bioscience #BD610418). Secondary species-specific HRP-conjugated antibodies (BioRad) have been used. Signals were visualized using the chemiluminescent reagent ECL (GE Healthcare) or LiteAblot TURBO (EuroClone) on an Uvitec Mini HD9 apparatus (Eppendorf). The intensity of the bands was analyzed using ImageJ software (US National Institutes of Health) and GFP signal was normalized to HSP90.

### Mouse Dimension and Preparation of Mouse Tissues

The mouse length was measured from the nose to the back of P13 and P75 individuals, using a ruler.

P0 mice ubiquitously expressing mt-Cam were identified upon illumination with a Dual Fluorescent Protein Flashlight lamp (Nightsea). Fluorescent pups were killed by decapitation, brain was extracted, rapidly cut at the vibratome (Leica, VT 1200S) in PBS and visualized by confocal microscopy (Leica TCS SP5 II confocal system).

Adult mice (> P30) were killed with an overdose mixture of Zoletil (30 mg·kg^−1^) and Xylazine (5 mg·kg^−1^) and transcardially perfused with 0.9% NaCl isotonic saline followed by paraformaldehyde (PFA) 4% in PBS. Organs were collected, cryoprotected in sucrose solution (30%) for 5–6 days, included in PolyFreeze (Tissue Freezing Medium)—Clear (Polysciences, #19636-1) and cut (20 μm, coronal sections) at the cryostat (Leica, CM 1850) or post-fixed in PFA 4% at 4°C O/N, included in agarose low gelling temperature (Sigma-Aldrich, #A9414) and cut (60 µm, coronal or sagittal sections) at the vibratome (Leica, VT12005). Collected slices were mounted, coverslipped with Mowiol, and imaged at the confocal microscope (Leica TCS SP5 II confocal system).

### Immunofluorescence on Tissue Slices

Sections fixed as described above were blocked with 0.5% Triton X-100 + 5% normal donkey serum in PBS for 1 h at RT, then incubated with rabbit anti-TOM20 (1:50, Santa Cruz Biotechnology, #sc-11415), mouse anti-NeuN (1:1000, Sigma-Aldrich, #MAB377), or rabbit anti-GFAP (1:500, Dako, #Z0334) O/N at 4°C. After rinsing, sections were incubated with donkey anti-rabbit or anti-mouse Alexa 555 (1:500, Thermo Fisher Scientific, #A31572 and #A31570) for 2 h at RT.

To mark nuclei, fixed slices were incubated with the nuclear marker TO-PRO 3 (1:10000, Sigma-Aldrich) for 10 min at RT and rinsed in PBS.

Slices were mounted and coverslipped with Mowiol, then imaged using a Leica TCS SP5 II confocal system equipped with a HCX PL Fluotar 20×/0.50 or a HCX PL APO 100×/1.40 objective. The Argon laser line (488 nm) was used to excite fluorescent proteins (FPs) of the Cameleon sensor. Confocal microscopy imaging was performed at 1024 × 1024 pixels per image, with a 100 Hz acquisition rate, average of two frames.

The analysis of the area occupied by mitochondria expressing the probe was performed on 3 slices from 3 animals using ImageJ software (US National Institutes of Health). For each image, a threshold was imposed and the fluorescent area was determined.

### Primary Cultures from the Brain of Newborn Mice

Primary cortical and hippocampal neuronal cultures were obtained from P0/P1 mice. Tissues from five pups were dissociated in trypsin (0.8 mg·mL^−1^) for 10 min at 37°C and digestion was stopped by adding FBS (Euroclone) and DNAse I (40 μg·mL^−1^). The pellet obtained by centrifugation (1400 rpm for 5 min) was suspended in MEM YUE medium, containing MEM (Gibco, #32360-026), 10% horse serum, 20 mM glucose, 1 mM sodium pyruvate, 0.875 μg·mL^-1^ Biotin, 0.5 mM L-Glutamine, 0.5% B-27 (Thermo Fisher Scientific), 1% N-2 (Thermo Fisher Scientific), 100 U·mL^−1^ penicillin, and 100 μg·mL^−1^ streptomycin, then plated on glass coverslips (18 mm) coated with poly-L-lysine (100 µg·mL^−1^) for cortical or poly-L-lysine (30 µg·mL^−1^) and laminin (2 µg·mL^−1^) for hippocampal neurons at a density of 0.6 × 10^6^ cells/coverslip. After 24 h, the growth medium was replaced with serum- and antibiotic-free Neurobasal-A medium (Thermo Fisher Scientific, #10888022), containing 2% B-27 (Thermo Fisher Scientific), 0.5 mM L-Glutamine (Thermo Fisher Scientific), and 3 µM cytosine arabinoside. Fresh medium was added (1/5 of total volume) every fifth day. Neurons were imaged after 7 days *in vitro* (DIV).

Cortical astrocytes were isolated using the same dissociation protocol described above for cortical neurons and plated at a density of 0.2 × 10^6^ cells/coverslip on glass coverslips (18 mm) coated with poly-L-lysine (100 µg·mL^−1^). Twenty-four hours after dissociation, the growth media was replaced with DMEM with high glucose (Sigma-Aldrich, #5671) with the addition of FBS 10%, L-Glutamine 0.5 mM, penicillin 100 U·mL^−1^, and streptomycin 100 μg·mL^−1^. Astrocytes were imaged after 7 DIV.

### Flexor Digitorum Brevis Fibers Isolation

Flexor digitorum brevis (FDB) muscles were dissected from P60 mice. Muscles were enzymatically digested in Tyrode’s salt solution (Sigma-Aldrich, #T2145) supplemented with 10 mM HEPES, 10% FBS (Thermo Fisher Scientific, #16250078), 5.5 mM glucose, and 4 mg·mL^-1^ collagenase A (Roche, #10103586001) (1 h at 4°C to allow the penetration of Collagenase A into the tissue, then 45–50 min at 37°C to optimize Collagenase A action). Then, muscles were mechanically dissociated in DMEM with HEPES (Thermo Fisher Scientific, #42430) supplemented with 10% FBS, penicillin (100 U·mL^−1^), and streptomycin (100 μg·mL^−1^). Dissociated fibers from each FDB were plated on 12 glass coverslips (24 mm) pre-coated with laminin (Roche, #11243217001) and maintained O/N at 37°C with 5% CO_2_.

### Primary Culture Staining

Neurons and astrocytes plated on coverslips were immune-labeled with mouse anti-NeuN (1:1000, Sigma-Aldrich, #MAB377) or rabbit anti-GFAP (1:500, Dako, #Z0334), respectively. The signal was revealed by using secondary antibodies donkey anti-rabbit or anti-mouse Alexa 555 (1:500, Thermo Fisher Scientific, #A31572 and #A31570).

Alternatively, plated neurons, astrocytes or fibers were loaded with the cell-permeant dye Tetramethylrhodamine, methyl ester (TMRM, Molecular Probes), 20 nM for 20 min at 37°C.

Stained neurons, astrocytes or fibers were imaged at a Leica TCS SP5 II confocal system, equipped with a PlanApo 100×/1.4 objective. The Argon laser line (488 nm) was used to excite FPs of the Cameleon sensors and the He/Ne 543 nm laser was used to excite the Alexa 555 secondary antibody. Confocal microscopy imaging was performed at described above.

### *In Vitro* Ca^2+^ Imaging

Ca^2+^ imaging experiments were performed on plated hippocampal and cortical neurons or astrocytes at 7 DIV with an inverted microscope (Nikon Ti-E) equipped with an S fluor DIC H N2 40×/1.30 oil objective. Excitation at 425 nm was obtained with a 75 W Xenon Lamp (USHIO, UXLS50A) and an Optoscan monochromator (CAIRN-Research) controlled by NIS-E software (Nikon). An FF-458-Di02 dichroic mirror (Semrock) was used in the excitation pathway. The emitted fluorescence was collected through an Optosplit beam splitter (CAIRN-Research) equipped with FF01-479/40 and FF01-542/27 emission filters (Semrock) for enhanced cyan fluorescent protein (ECFP) and circularly permuted Venus (cpV), respectively, and a T515LPXR-UF2 dichroic mirror (Chroma). Images were acquired at 1 Hz with 100 ms exposure time by a Zyla-CMOS 4.2-P camera (Andor, Oxford Instruments). The coverslips were mounted into an open-topped chamber (RC-41LP, Warner Instruments) and maintained in a modified Krebs-Ringer buffer (KRB) solution of the following composition (in mM): NaCl 140, KCl 2.8, MgCl_2_ 2, HEPES 10, CaCl_2_ 1, glucose 5, pH 7.4 at RT.

Cells were challenged with the following stimuli: ATP (100 µM, Sigma-Aldrich, #A2383), N-methyl-D-aspartate (NMDA) (20 µM, Sigma-Aldrich, #M3262), glycine (40 µM, Sigma-Aldrich, #G8898), (*S*)-3,5-Dihydroxyphenylglycine (DHPG) (100 µM, Abcam, #ab120007), carbachol (300 µM, Sigma-Aldrich, #C4382), glutamate (100 µM, Sigma-Aldrich, #G1251). Where indicated, ethylene glycol-bis(β-aminoethyl ether)-N,N,N′,N′-tetraacetic acid (EGTA) (1mM manually added or 0.5 mM perfused, Sigma-Aldrich, #03777) or Carbonyl cyanide-4-(trifluoromethoxy)phenylhydrazone (FCCP) (2 µM, Sigma-Aldrich, #C2920) were added.

For FDB fibers, real-time imaging was performed the day after plating using a DM6000 inverted microscope (Leica, Wetzlar, Germany) with a HCX Plan Apo 40×/1.25 oil objective. The excitation light, produced by a 405 nm LED (LED Engin, #LZ1-00UA00) was filtered by an ET410/20 band pass filter and 455 dichroic mirror. The emitted light was collected through a custom-made beam splitter (ET480/40 and ET535/30 emission filters for ECFP and cpV, respectively, and a 515DCXR dichroic mirror (Chroma). All filters and dichroic mirrors were from Chroma Technologies. Images were acquired using an IM 1.4C cool camera (Jenoptik Optical Systems) attached to a 12-bit frame grabber. Synchronization of the excitation source and the cool camera was obtained through a control unit run by custom-made software package Roboscope (developed by Ciubotaru C. D., Padua, Italy). Images were acquired at 1 Hz with 100–200 ms exposure time. The coverslip was mounted into an open-topped chamber and maintained in a modified KRB solution of the following composition (in mM): NaCl 135, KCl 5 , MgCl_2_ 1, HEPES 20,  MgSO_4_ 1, KH_2_PO_4_ 0.4, CaCl_2_ 1, glucose 5, pH 7.4 at RT, in the presence of 75 μM BTS (N-benzyl-P-toluene sulphonamide) (TOCRIS, #1870) to avoid fiber contraction. In a typical experiment, after recording basal Ca^2+^ level, caffeine (20 mM, Sigma-Aldrich, #C0750) was added to elicit Ca^2+^ release from intracellular stores.

### Two Photon Ca^2+^ Imaging on Acute Brain Slices

Mice from P20 to P40 were killed with an Xylazine–Zoletil overdose and transcardially perfused with an artificial cerebrospinal fluid (ACSF) solution (in mM, NaCl 125, NaHCO_3_ 26, KCl 3, NaH_2_PO_4_ 1.25, glucose 20, MgCl_2_ 1.3, CaCl_2_ 2, pH 7.4 at RT). Brains were removed and sectioned (300 μm, coronal sections) at the vibratome (Leica, VT 1200S). The entire procedure was performed with the brain submerged in ice-cold Cutting Solution (in mM, sucrose 210, NaHCO_3_ 26, KCl 3, NaH_2_PO_4_ 1.25, glucose 20, MgCl_2_ 3, CaCl_2_ 0.5) and bubbled with carbogen (95% O_2_ and 5% CO_2_). Slices were then transferred in a chamber containing ACSF, incubated at 32°C for 30 min and then maintained at RT for 30 min. Imaging was performed with a two-photon laser-scanning microscope (Scientifica Ltd) equipped with a Nikon LWD 16×/0.8 water immersion objective and a laser system operating at 850 nm (power at the objective 12–24 mW). Fluorescence emissions were collected using two photomultipliers (PMT 1 set at 684 nm; PMT 2 set at 714 nm) through a custom made beam splitter (ET480/40 and ET535/30 emission filters; T510LPXRXT dichroic mirror (Chroma)). Brain slices were placed in an open chamber perfused with ACSF solution at 32°C continuously bubbled with carbogen (95% O_2_ e 5% CO_2_) at 2 mL·min^−1^. Stimuli were added also through perfusion: ATP (100 µM), DHPG (100 µM), carbachol (300 µM), and glutamate (100 µM). The acquisition rate was 1.72 frames·s^−1^ and the size of the imaging field was 512 × 512 pixels. Images were analyzed off-line with ImageJ software (US National Institutes of Health).

### Data Analysis and Statistics

Off-line analysis of Ca^2+^ imaging experiments was performed using ImageJ software (US National Institutes of Health). Proper regions of interest (ROIs) were selected for each image and fluorescence signals *F*_cpV_ and *F*_ECFP_ were subtracted (*in vitro* experiments) or not (*ex vivo* experiments) of background. The ratio of the emitted fluorescence intensities (*R* = *F*_cpV_/*F*_ECFP_) was calculated for each ROI and each timeframe. Data are presented as the relative change in *F* or *R* normalized to the value measured before stimulus addition (Δ*F*/*F*_0_; Δ*R*/*R*_0_).

Data were analyzed using Microsoft Excel. Values are expressed as mean ± SEM with *n* = number of animals in immunohistochemistry, or SD with *n* = number of lanes loaded for western blotting. Statistical analyses were performed using unpaired Student’s *t*-test with a confidence interval of 95% (*p < 0.05, **p < 0.01, and ***p < 0.001).

## Results

### Generation of the Transgenic ROSA26-mt-Cam Mouse Line

In our effort to improve current strategies for *in vivo* expression of Ca^2+^ sensors, we developed a transgenic mouse line for the controlled expression of a mitochondria-targeted probe. Among others, we selected the FRET-based Cameleon 4mtD3cpv because it allows ratiometric imaging, with the great advantage of reducing sensitivity to motion artifacts and probe expression levels. To avoid toxicity due to constitutive expression of the exogenous protein and to guarantee the possibility of accessing specific cell types using selected promoters, we exploited a well-established Cre/*loxP* recombination system.^63^

We combined this approach with site-directed transgenesis at the *ROSA26* locus, in order to prevent the issue of unpredictable expression due to the effect of random genomic integration site or transgene copy number.^64^ Indeed, this site provides high targeting efficiency and transgene transcription at adequate levels.^60^^,^^65^ When directed by a CAG promoter, expression is observed in all organs/tissues analyzed, although the proportion of positive cells substantially varies among them.^59^^,^^66^ A MultiSite Gateway approach was exploited to build a conditional *ROSA26*-targeting vector for transgenesis.^59^ The vector consisted of the CAG promoter^67^*floxed* βgeo-STOP cassette^59^ followed by mt-Cam, a codon-optimized version of 4mtD3cpv cDNA sequence. The obtained targeting vector ensures the insertion of mt-Cam sequence into the *ROSA26* locus in anti-sense orientation, relative to the *ROSA26* promoter (Figure S1A). Anti-sense orientation was chosen because it was previously shown to yield higher levels of expression, compared to the sense.^68^ The vector was used for ES cell electroporation. Correct *ROSA26* locus targeting was screened by Southern blot analysis of genomic DNA extracted from surviving colonies using 5′ (550 bp) and 3′ (800 bp) external probes together with an internal probe designed on mt-Cam sequence (800 bp) to confirm 5′- and 3′-integration, as well as single-copy insertion (Figure S1B). Correctly targeted ES cells were injected into host blastocysts of C57BL/6J mice, which were re-implanted into pseudo-pregnant Swiss Webster females. Chimeric animals were mated to C57BL/6J mice to generate heterozygous germline offspring. Resulting offspring were genotyped to check for the presence of mt-Cam sequence.

### Ubiquitous Expression of mt-Cam

**Figure 1.**
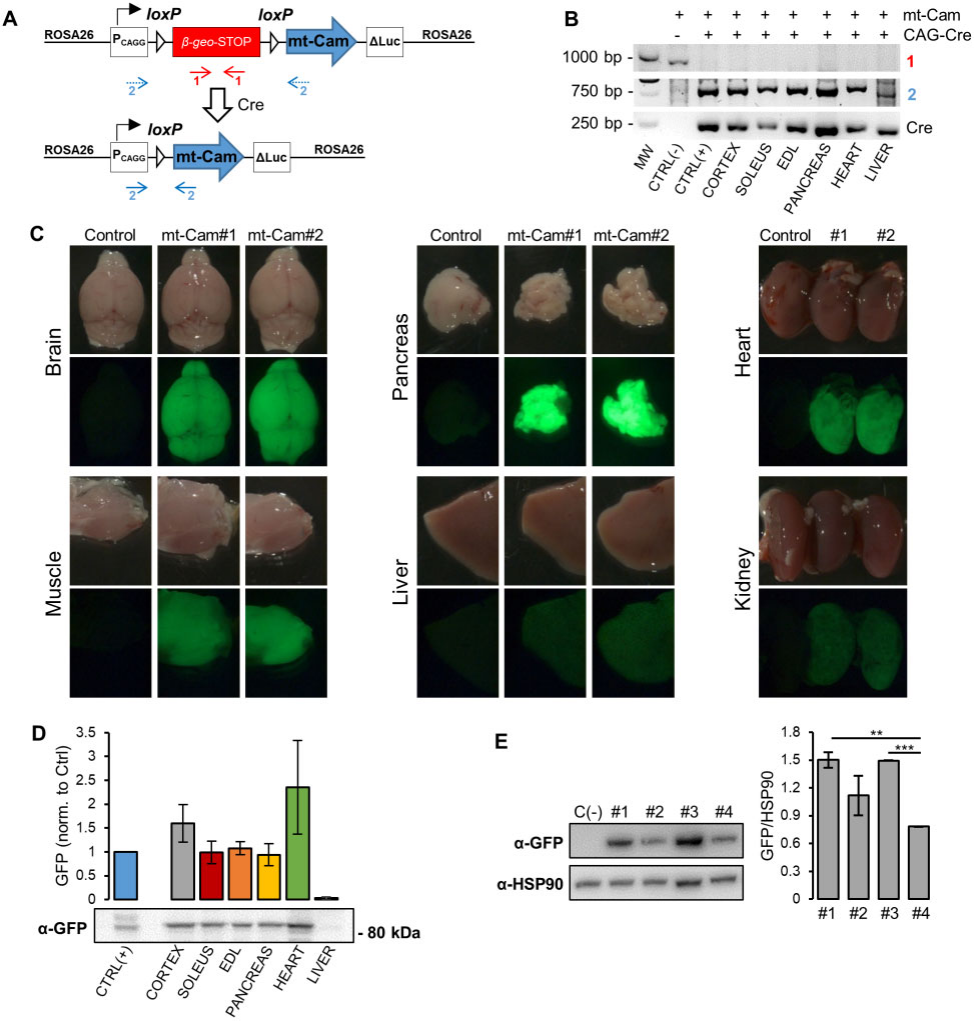
Generation of a Cre/*loxP* conditional transgenic mice for the expression of mitochondria-targeted Cameleon probe 4mtD3cpv. (**A**) Schematic of the mt-Cam sequence inserted in *ROSA26* genomic region, before (top) and after (bottom) Cre-mediated excision. (**B**) PCR analysis of the indicated tissues extracted from a mt-Cam-expressing mouse confirmed the excision of the *loxP*-STOP-*loxP* in the presence of ubiquitous CAG-Cre. PCR reactions were performed using primers annealing within the *loxP*-STOP-*loxP* region (region “1”, red, as depicted in **A**) or external to the *loxP*-STOP-*loxP* region (region “2”, blue, as depicted in **A**). PCR amplifying Cre sequence confirmed the ubiquitous presence of Cre recombinase (lower lane). Controls are derived from mice positive for both mt-Cam and CAG-Cre (positive control, expressing mt-Cam) or only mt-Cam (negative control), respectively. In negative control the *loxP*-STOP-*loxP* region is not excised. (**C**) Bright-field and fluorescence images of the organs indicated, collected from two mt-Cam-expressing animals and a littermate negative control. (**D**) Western blotting analysis of tissue lysates from the mt-Cam mice, obtained as described in panel B. The histogram represents the quantification of α-GFP antibody signal, which also detects ECFP, normalized to a control sample obtained from HeLa cells transfected with a plasmid encoding 4mtD3cpv (mean ± SEM, *n* = 3). (**E**) Western blotting analysis of cortex lysates from mt-Cam-expressing mice (#1 to #4) and a negative control. The histogram represents the quantification of α-GFP antibody signal normalized to the housekeeping protein HSP90 (mean ± SEM, *n* = 3; **p < 0.001; ***p < 0.001).

The ROSA26-mt-Cam mice obtained were crossed with mice ubiquitously expressing Cre recombinase (CAG-Cre mice^62^; Figure S1C) and positive offspring was selected by PCR (Figure S1D). PCR analysis on genomic DNA confirmed the complete excision of the βgeo-STOP cassette in male and female offspring positive for both mt-Cam and Cre (Figure 1A and B). Noteworthy, the probe is expressed in early stages of development and it is easy to score also in intact P0-P1 pups observed under a fluorescence lamp or microscope (Figure 1E). However, when observed at the fluorescence microscope, high variability of fluorescence intensity level was observed among animals, even siblings (Figure 1E; Figure S1E). Moreover, we observed that the expression of the probe is variable in the different tissues analyzed (Figure 1C). Western blotting analysis of proteins extracted from different P30 organs confirmed the presence of variable amounts of the protein (Figure 1D). Significantly low expression was observed in some organs, for example, in the liver. The results were confirmed by fluorescence analysis of organ slices, showing that in liver sections the average area occupied by the fluorescent signals, which gives an estimate of the percentage of positive cells, was calculated as 0.9 ± 0.4% (mean ± SD) (Figure S2A and S2B), while in brain slices it was 18.5 ± 5.1% (mean ± SD) (Figure S2C and S2D). Differences in protein expression were recapitulated when mRNA relative expression was estimated by qPCR (Figure S1F) and are consistent among animals (data not shown).

**Figure 2.**
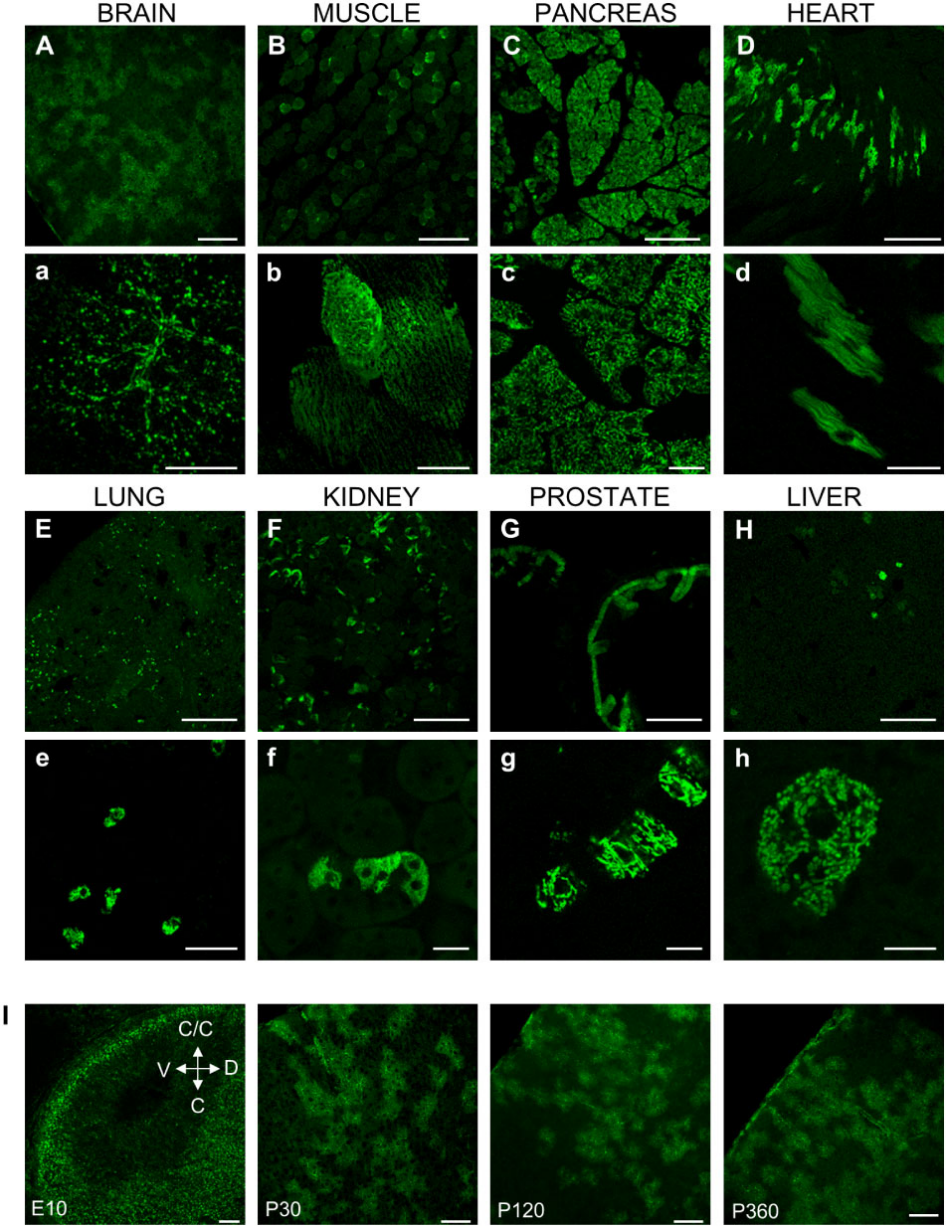
Expression of mt-Cam in mouse tissues. (**A–H**) Confocal images of sections of the indicated tissues (uppercase) and relative higher magnifications (lowercase) from P30 mice ubiquitously expressing mt-Cam. Scale bar 200 µm (A–J) or 20 µm (a–j). (**I**) Confocal images of coronal sections of embryonal (∼E10), postnatal day P30, P120 and P360 brains ubiquitously expressing mt-Cam. Abbreviations: C/C, cephalic/cranial; C, caudal; D, dorsal; V, ventral. Scale bar 100 µm.

Confocal imaging revealed the presence of positive fluorescent cells in all tissues/organs analyzed, *e.g.*, brain (Figure 2A-a), skeletal muscles (Figure 2B-b), pancreas (Figure 2C-c), heart (Figure 2D-d), lungs (Figure 2E-e), kidney (Figure 2F-f), prostate (Figure 2G-g), and liver (Figure 2H-h). Moreover, to check whether the expression of the probe is stable during development and aging, brain slices from mice at different ages, from embryonic stage E10 to P360, were analyzed at the confocal microscope. The expression of the probe is maintained during aging (Figure 2I).

**Figure 3.**
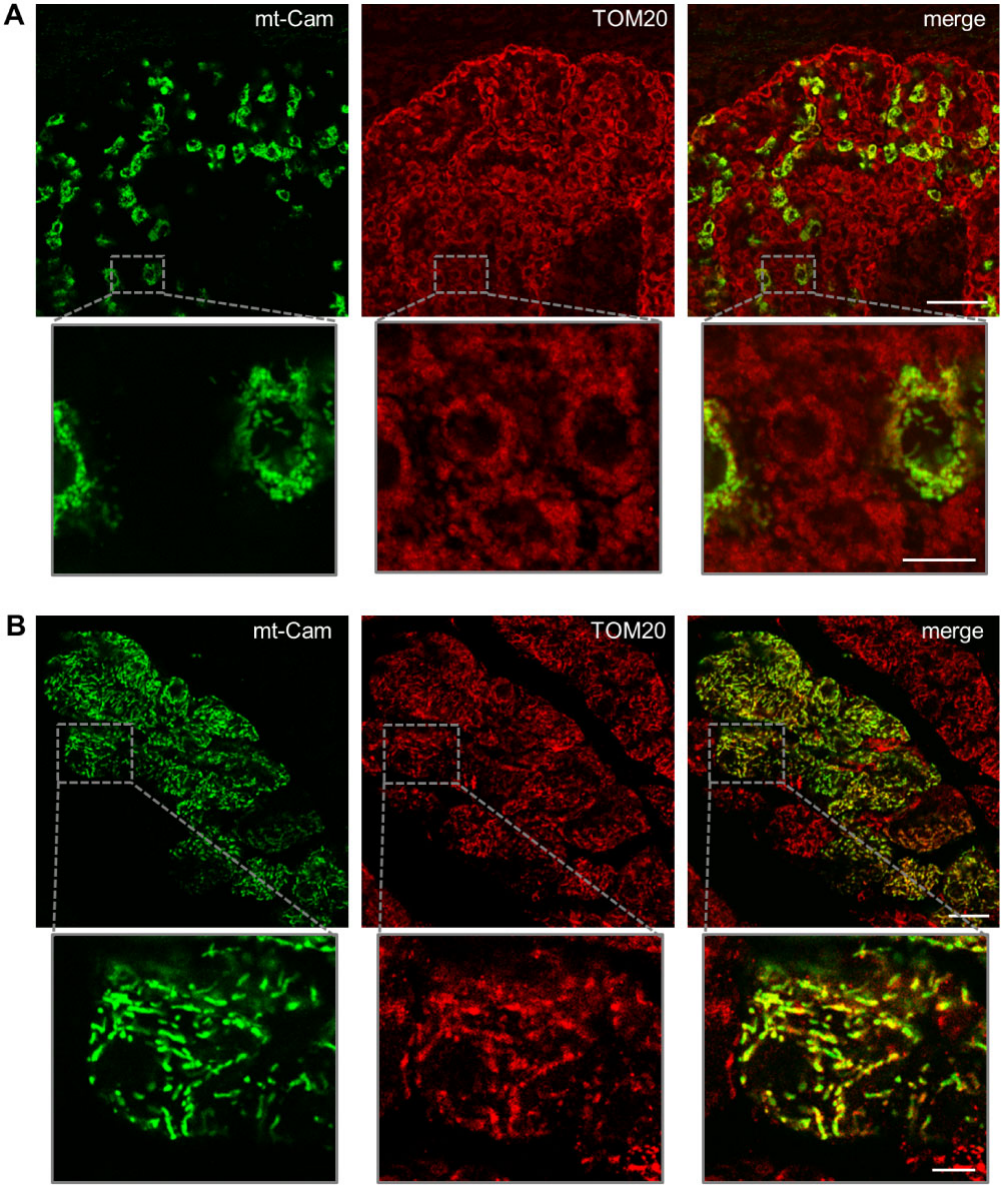
Mitochondrial localization of mt-Cam in brain and pancreas slices. Confocal microscope images of coronal sections of cortex (**A**) and pancreas (**B**) from a P120 mouse ubiquitously expressing mt-Cam (green), stained for TOM20 (red). Bottom panels show higher magnifications of the indicated regions (gray squares). Scale bar 75 µm or 15 µm (in higher magnifications).

The proper mitochondrial localization of the expressed probe was verified in the tissues analyzed. As an example, Figure 3 shows the immunohistochemical analysis of fixed pancreatic and brain slices stained with the mitochondrial marker TOM20. Noteworthy, the expression of the Cameleon probe does not alter the aspect and size of mice, which were similar to control littermates (Figure S1G).

### Mitochondrial Ca^2+^ Dynamics in the Central Nervous System of mt-Cam-expressing Mice

**Figure 4.**
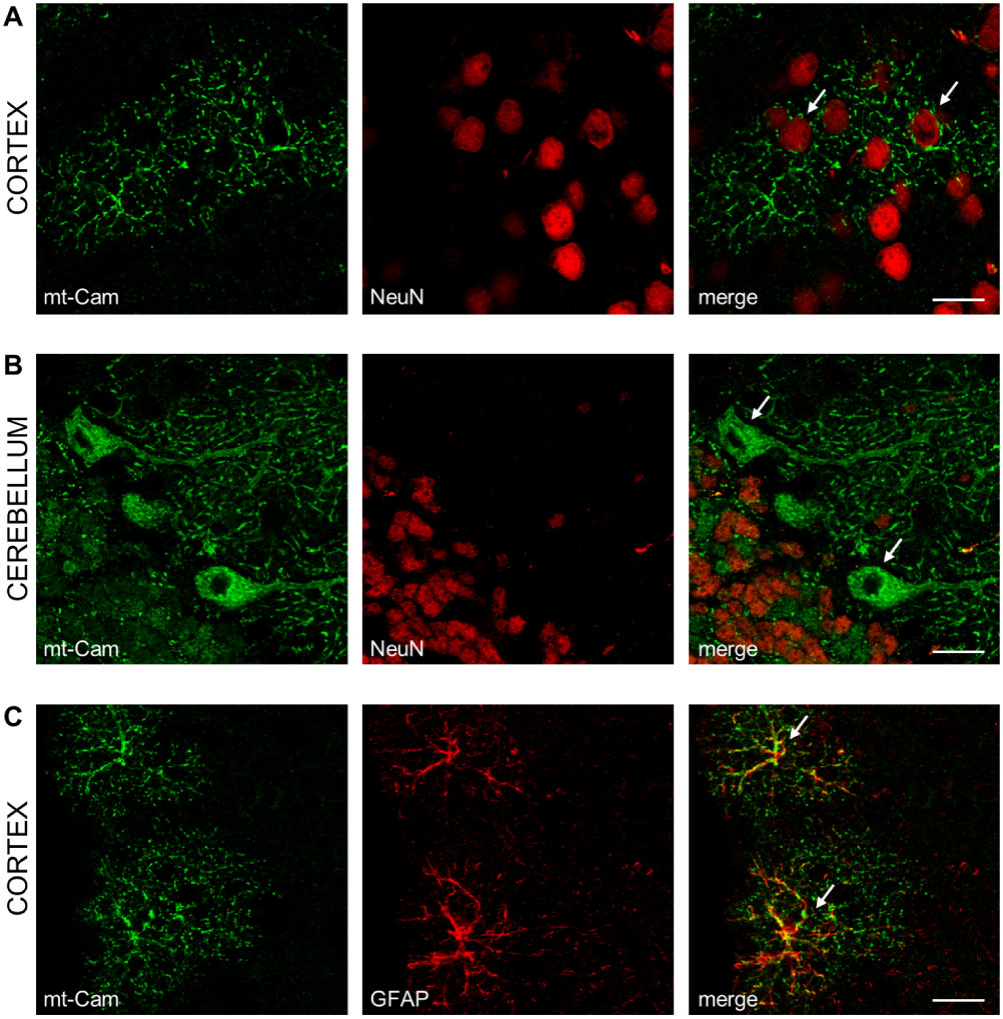
Confocal images of coronal sections of mouse brain expressing mt-Cam. Cortex (**A,C**) and cerebellum (**B**) from a mouse expressing mt-Cam (green), stained with the neuronal marker NeuN (**A,B**; red) or with the astrocyte marker GFAP (**C**; red). Note that Purkinje cells (arrows in **B**) are negative for NeuN staining.^69^ Scale bar 20 µm.

We then focused our analysis on the nervous system, where, at present, expression of mitochondria-targeted GECIs relies on viral infection.^39^ Immunohistochemical analysis of brain slices from positive animals, using neuron-specific NeuN or astrocyte-specific GFAP staining, revealed that both neurons and astrocytes expressed the fluorescent sensor (Figure 4). The Cameleon signal was clearly excluded from the nucleus (labeled by NeuN, in cortical neurons) and reached the finest processes (Figure 4).

**Figure 5.**
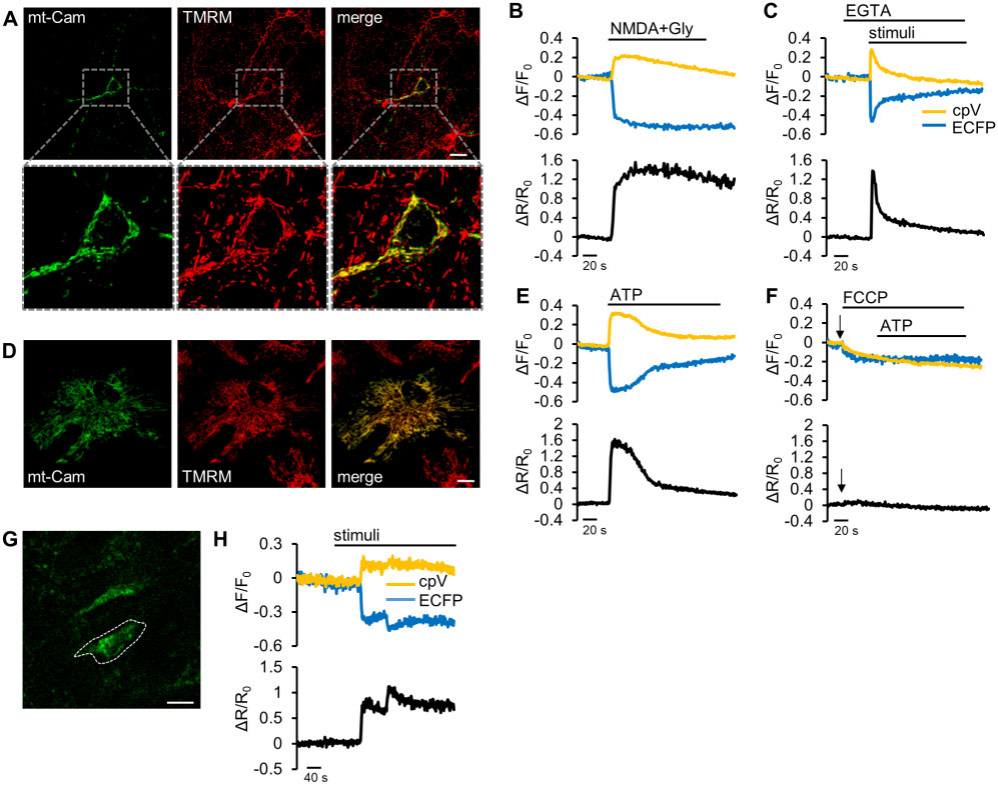
Expression and function of mt-Cam in neurons and astrocytes. (**A**) Confocal images of cortical neurons at 7 DIV, isolated from P0-P1 mice expressing mt-Cam (green), stained with TMRM (red); relative higher magnifications are shown in the insets. Scale bar 10 µm. (**B** and **C**) Representative traces obtained in mt-Cam-expressing mouse-derived cortical neurons upon stimulation with NMDA (20 µM) plus glycine (Gly, 40 µM) (**B**) or with a mix of stimuli: DHPG (100 µM), carbachol (300 µM), ATP (100 µM), glutamate (100 µM) in the presence of EGTA (1 mM) (**C**). Data are presented as: ΔF/F_0_ (top), ΔR/R_0_ (bottom). (**D**) Confocal images of cortical astrocytes at 7 DIV, isolated from P0-P1 mice expressing mt-Cam (green), stained with TMRM (red). Scale bar 10 µm. (**E and F**) Representative traces obtained in mt-Cam-expressing mouse-derived cortical astrocytes upon stimulation with ATP (100 µM) alone (**E**) or in the presence of the uncoupler FCCP (2 µM) (**F**). Data are presented as ΔF/F_0_ (top), ΔR/R_0_ (bottom). (**G and H**) Two-photon Ca^2+^ imaging on brain slices from P40 mt-Cam-expressing mouse. (**G**) Coronal section of mt-Cam expressing mouse brain imaged at the two-photon microscope. The circled ROI indicates the cell expressing mt-Cam probe analyzed in **H**. Scale bar 10 µm. (**H**) Representative traces obtained upon stimulation of the slice with a mix of stimuli as indicated in panel B, but in standard ACSF. Data are presented as ΔF/F_0_ (left), ΔR/R_0_ (right).

To investigate the ability of mt-Cam of reporting mitochondrial Ca^2+^ dynamics in neurons and astrocytes, we first set up a series of *in vitro* experiments. Primary neuronal cultures were prepared from the cortex or hippocampus of P0-P1 mice. The mitochondrial localization of mt-Cam was further confirmed by loading isolated cortical neurons (Figure S3A) with the cell-permeant dye TMRM, which is selectively sequestered by polarized mitochondria (Figure 5A). To verify whether ROSA26-mt-Cam mice express a functional probe, we performed Ca^2+^ imaging experiments on isolated hippocampal and cortical neurons. When Cameleon binds Ca^2+^, it undergoes a conformational change that increases FRET and, accordingly, we observe an increase in the 540 nm and a decrease in the 480 nm fluorescence (F) intensities, which result in an increased F_540_/F_480_ ratio. Both cortical (Figure 5A–C) and hippocampal neurons (Figure S4) were challenged with NMDA plus glycine to maximally activate NMDA receptors (Figure 5B; Figure S4A) or with a mix of stimuli, *i.e.* DHPG, carbachol, ATP, and glutamate (Figure 5C; Supplementary Figure 4B), in the absence of external Ca^2+^ and in the presence of EGTA, in order to induce maximal store depletion. In both cases a clear decrease of the ECFP emission signal (480 nm) and a concomitant increase of the cpV signal (540 nm) were observed (Figure 5B and C; Figure S4). In the latter experiment, the increase in the F_540_/F_480_ ratio is exclusively due to mitochondrial uptake of Ca^2+^ released from internal stores, thus resulting in a sharper peak, compared to that caused by NMDA plus glycine, whose Ca^2+^ rise is dominated by Ca^2+^ influx across the PM. After the peak, mitochondrial [Ca^2+^] returned rapidly to the basal level (Figure 5C;Figure S4B).

Similar results were obtained on primary cortical astrocytes isolated from P0-P1 animals. The presence of positive astrocytes is confirmed by the immunostaining with the specific marker GFAP (Figure S3B). TMRM loading confirmed the proper localization of the probe also in this cell type (Figure 5D). When stimulated with the purinergic agonist ATP, astrocytes showed the expected FRET response (Figure 5E), which was abolished if cells were pre-treated with the mitochondrial uncoupler FCCP (Figure 5F), further confirming the exclusive mitochondrial localization of the probe. A small drop is detected in ECFP and cpV fluorescence upon addition of FCCP (Figure 5F, upper panel, arrow), likely due to a change in pH and/or to mitochondrial swelling. Notably, this is efficiently corrected by ratio calculation (Figure 5F, lower panel, arrow).

To extend the validity of our approach, we analyzed mitochondrial Ca^2+^ dynamics of neurons in acute brain slices. We performed *ex vivo* two-photon Ca^2+^ imaging experiments in coronal slices of P40 mt-Cam-expressing mice (Figure 5G). After recording the basal ratio at the cortical level, we perfused a mix of stimuli (ATP, DHPG, carbachol, and glutamate) aimed at activating both ionotropic and metabotropic receptors, thus triggering major mitochondrial [Ca^2+^] increases. Again, Ca^2+^ entry into the mitochondrial matrix was readily revealed by a decrease of the ECFP signal and a concomitant increase of the cpV signal, causing an increase in F_540_/F_480_ ratio R (Figure 5H).

Finally, as a proof of principle example of tissue-specific expression of the probe, ROSA26-mt-Cam mice were crossed with ChAT-Cre mice, expressing Cre recombinase under the control of the ChAT promoter. Analysis of sagittal brain sections confirmed the presence of clusters of cholinergic neurons expressing the mt-Cam probe, mostly localized in the midbrain, close to the cerebellum (Figure S5).

### Mitochondrial Ca^2+^ Dynamics in FDB Muscle Fibers Isolated from mt-Cam Mice

Increases in mitochondrial matrix [Ca^2+^] are crucially important in skeletal muscle where, by stimulating Ca^2+^-sensitive key enzymes such as pyruvate dehydrogenase,^19^^,^^20^^,^^70^ they ensure the optimal production of ATP enabling the cross-bridge cycle of actomyosin. Currently, the use of GECIs to measure mitochondrial Ca^2+^ in fibers requires great expertise, since it relies on *in vivo* plasmid DNA electroporation.

**Figure 6.**
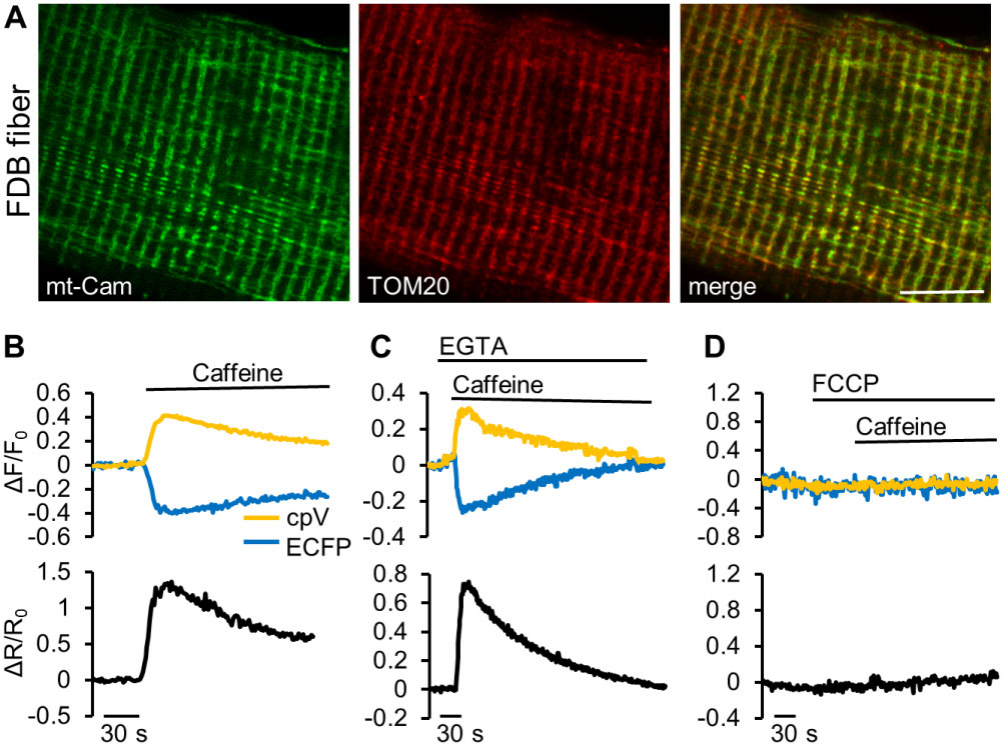
Ca^2+^ imaging in FDB fibers dissociated from mt-Cam expressing mice. (**A**) Confocal images of a dissociated skeletal muscle fiber expressing mt-Cam (green), immunostained for TOM20 (red). Scale bar 10 µm. **(B–D)** Representative traces of Ca^2+^ dynamics in a fiber upon stimulation with caffeine (20 mM) in extracellular-like saline containing 1 mM Ca^2+^ (**B**) or without Ca^2+^ and in the presence of 0.5 mM EGTA (**C**). (**D**) As in **B**, but in the presence of the uncoupler FCCP. Data are presented as: ΔF/F_0_ (top), ΔR/R_0_ (bottom).

When ROSA26-mt-Cam mice are crossed with CAG-Cre mice, 4mtD3cpv is expressed in both cardiac and skeletal muscles in the offspring (Figure 2). We thus isolated FDB muscle fibers from mt-Cam expressing mice and verified the correct mitochondrial localization with anti-TOM20 antibody staining (Figure 6A). In the isolated muscle fibers, probe functionality was assessed by applying to the fibers caffeine, a ryanodine receptor agonist, in order to induce Ca^2+^ release from the SR, rapidly triggering mitochondrial Ca^2+^ uptake (Figure 6B). The same type of experiment was then carried out in the absence of extracellular Ca^2+^ and in the presence of EGTA, thus avoiding the influx of Ca^2+^ across the PM (Figure 6C). When cells were pre-treated with FCCP, the mitochondrial response to caffeine stimulation was completely abolished (Figure 6D).

## Discussion

The pivotal role of Ca^2+^ signaling in regulating central aspects of physiology and pathology, as well as the importance of mitochondria in this signaling, is widely accepted.^13^^,^^20^^,^^23^^,^^25–29^^,^^71^ Mitochondrial Ca^2+^ dynamics can now be routinely studied in cell cultures thanks to an extended palette of recently developed chemical^35^ and genetically encoded probes.^4^^,^^72^^,^^73^ In contrast, only a few transgenic mouse lines have been developed expressing mitochondria-targeted Ca^2+^ sensors.^53^^,^^54^ These latter are nonratiometric, bioluminescence-based sensors that suffer from low spatial resolution and are not suitable to perform single cell mitochondrial Ca^2+^ imaging. As a result, *in vivo*/*ex vivo* experiments still rely on viral injection or *in situ* electroporation, with obvious drawbacks such as limited access to areas of interest, laborious procedures, and potential toxicity.

Single fluorophore-based indicators GCaMPs are widely used to study Ca^2+^ dynamics in living organisms and several transgenic mouse line expressing cytosolic versions of these probes have been created^46^^,^^48^ (https://www.jax.org; https://www.janelia.org). Although the dynamic range of GCaMPs is in general higher, FRET-based indicators appear superior to correct movement artifacts, photobleaching, pH sensitivity and, most relevantly, they allow the quantitative estimation of [Ca^2+^]. The insertion of a FRET-based Cameleon probe in the *ROSA26* locus under the control of a Cre/*loxP* expression system, allowed the generation of mice expressing a FRET-based probe upon a single cross leading to Cre-mediated excision. The great potential of Cre/*loxP* expression system resides in the possibility of controlling the transgene expression in a temporal and cell-specific manner.^63^ Indeed, the transgenic model we generated allows the expression of a mitochondria-targeted Ca^2+^ sensor in regions otherwise difficult to inject/electroporate, such as deep brain structures or pancreas, upon crossing with a transgenic mouse expressing Cre recombinase under the control of a generic or cell-specific promoter.

As a proof of principle of the functionality of the tool generated, a ubiquitous Cre (CAG-Cre) was exploited to drive the expression of the Cameleon probe. The protein was detected in all the tissues/organs analyzed, throughout development and aging, confirming a widespread and stable expression of the probe. The CAG promoter is one of the most active promoters^74^; however, its high variability in transgene expression *in vivo* has been reported,^75^ even when inserted in the *ROSA26* locus.^59^^,^^66^ Our results confirm this observation, indeed we found an uneven expression of the probe within the analyzed tissues and among animals, even if littermates.

The expressed Cameleon is correctly localized in mitochondria of tissues from P0 to adulthood. Two-photon Ca^2+^ imaging experiments in *ex vivo* brain slices demonstrated the proper functionality of the probe. Moreover, we also demonstrated the feasibility of the generated tool for *in vitro* imaging. Indeed, Ca^2+^ imaging on primary cultures is often challenging due to poor transfection of some dissociated cells (*e.g.*, neurons) or the need of electroporation (*e.g.*, muscle fibers). Here we show that cultured neurons or astrocytes from newborn mice and muscle fibers from adult mice display a robust expression of the Cameleon probe and can thus be readily imaged without further procedures, that is viral infection. Noteworthy, the decay of matrix Ca^2+^ transient we recorded after ATP stimulation of isolated astrocytes is similar to that previously observed in isolated astrocytes transfected with 4mtD3cpv,^76^ suggesting the absence of adaptive effects on matrix Ca^2+^ dynamics arising in the transgenic animal. Crossing with cholinergic neuron-specific ChAT-Cre mice demonstrated that ROSA26-mt-Cam mice can easily be used for studying specific subpopulations of cells, upon crossing with any specific Cre mice line of choice.

We created the first line of transgenic mice of our knowledge that allows expression in a cell type of choice of a ratiometric Ca^2+^ probe, targeted to mitochondria. The great advantage of our system is its high versatility: Cre recombinase expression under the control of specific promoters allows the expression of the probe in virtually any tissue or cell type, ensuring the possibility of performing *in vitro*, *ex vivo*, and *in vivo* Ca^2+^ imaging experiments (alone or in combination with other techniques, *e.g.*, electrophysiology, optogenetics), both in physiological and pathological conditions.

## Supplementary Material

Supplementary material is available at the *APS Function* online.

## Supplementary Material

zqab012_Supplementary_Data
