# Supplementary material for: A New Transgenic Mouse Line for Imaging Mitochondrial Calcium Signals
Source: Function (Oxf). 2021 Feb 25;2(3):zqab012. doi: 10.1093/function/zqab012 (PMC8788866; doi:10.1093/function/zqab012)
Supplement: zqab012_Supplementary_Data [file zqab012_supplementary_data.docx]

**Supplementary Figures:**

**
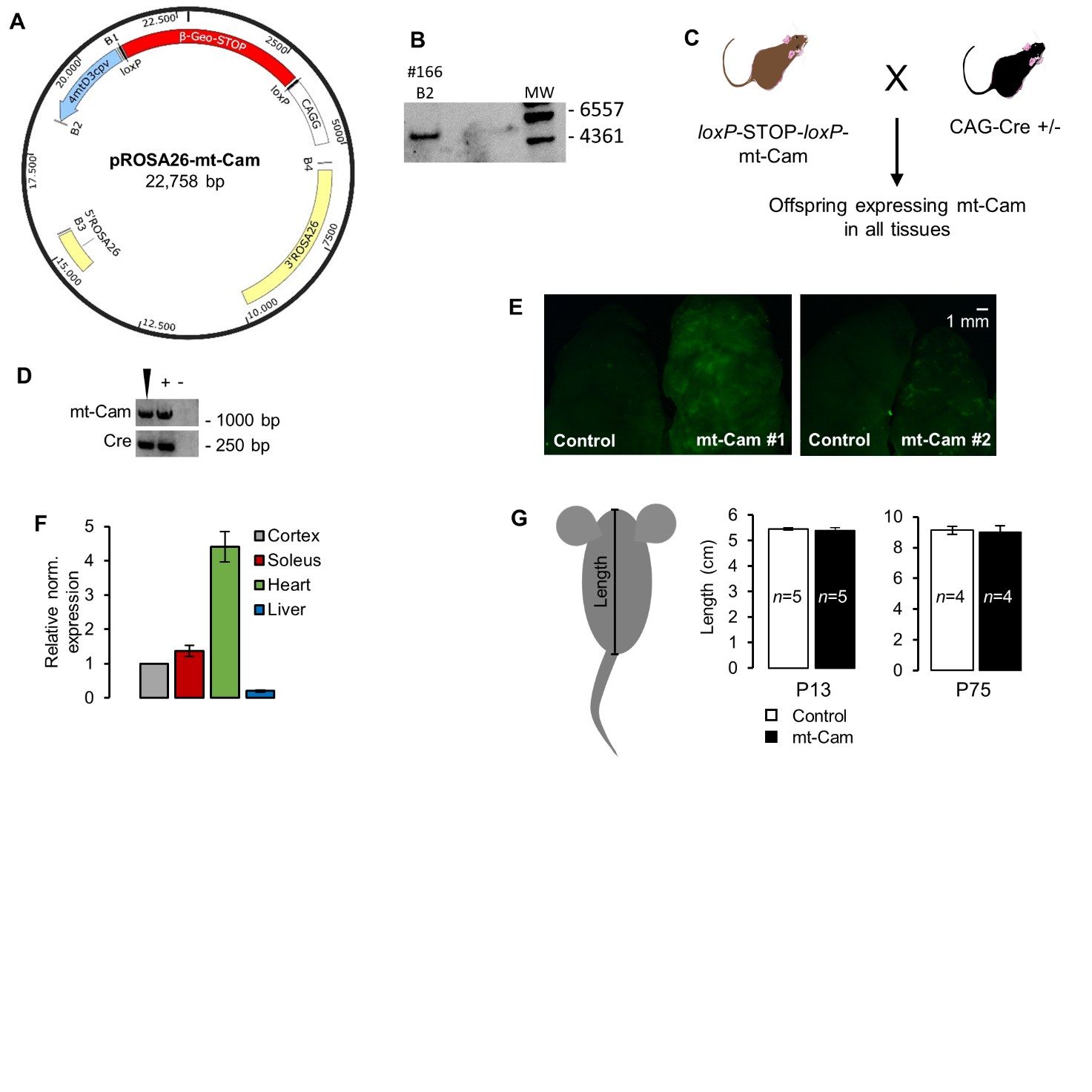
**

**Supplementary Figure 1. Generation of mt-Cam-expressing mice**. (**A**) Schematic of the construct used to generate ROSA26-mt-Cam transgenic mice. (**B**) Southern Blot analysis confirmed the correct integration of a single copy of the transgene. An internal probe on Cameleon sequence recognized a single band of 4900 bp. (**C**) Mice generated from *ROSA26*-targeted ES cells are crossed with CAG-Cre mice to generate transgenic offspring ubiquitously expressing mt-Cam. (**D**) PCR analysis on the offspring confirmed the presence of mt-Cam (up, 1660 bp) and Cre (bottom, 230 bp) sequences in the offspring of the cross described in C. (**E**) Fluorescence microscope images of two P0 mice expressing the mitochondria-targeted Cameleon probe and their control littermates. (**F**) Quantification of 4mtD3cpv mRNA in the tissues indicated, extracted from the floxed mt-Cam mouse described in Figure 1B,D, relative to TOM20 mRNA. Data are presented as mean ± SD, n = 3 plates. (**G**) Mean length of P13 and P75 mt-Cam mice compared to littermate controls, measured as indicated. n = number of animals.


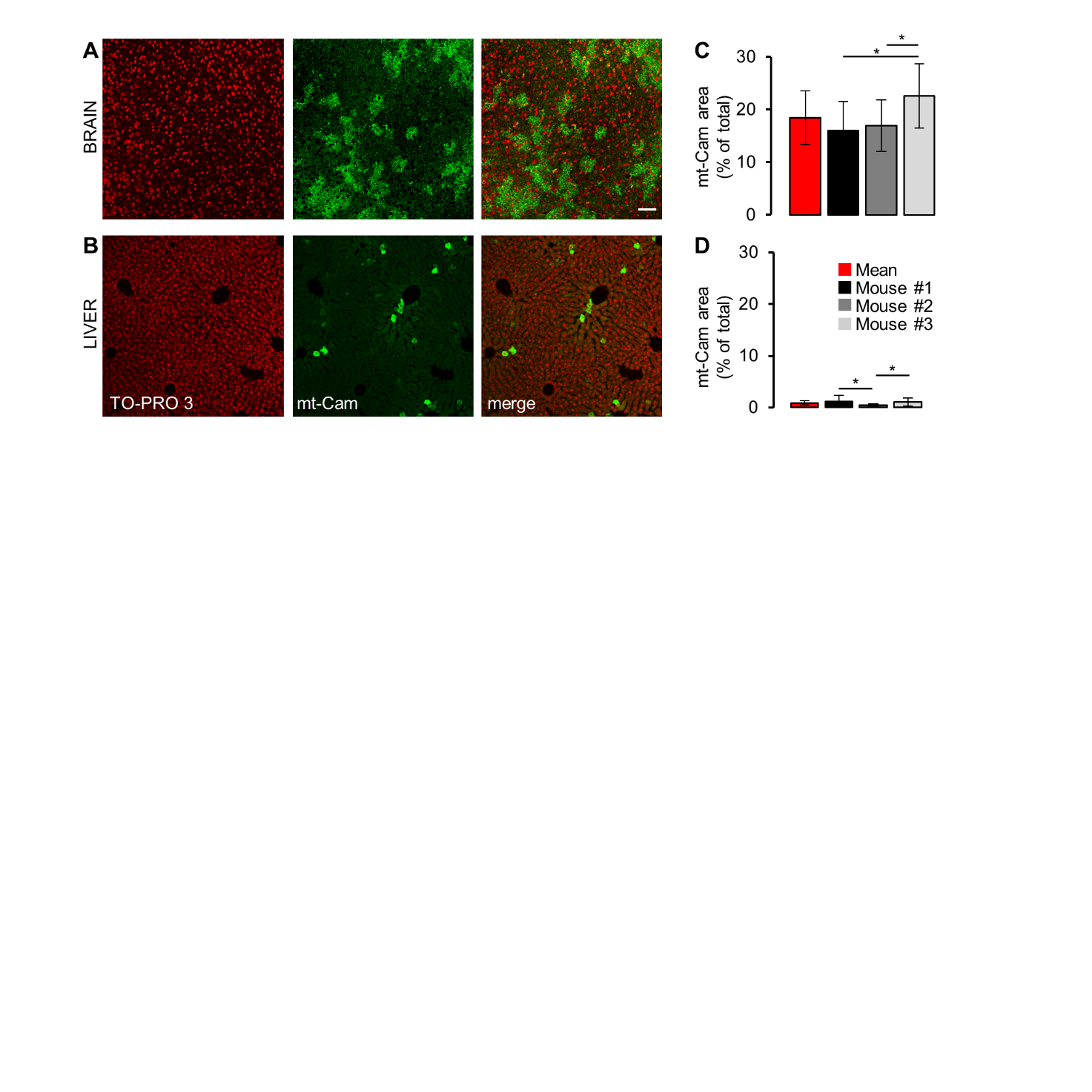


**Supplementary Figure 2**. **Evaluation of the amount of mt-Cam positive cells in brain and liver.** (**A-B**) Brain (**A**) and liver sections (B) from animals expressing mt-Cam were stained with the nuclear marker TO-PRO (red). The area occupied by mt-Cam signal (green) was quantified. Scale bar 100 µm. (**C-D**) Histograms showing the mean percentage of imaged area occupied by mt-Cam signal in the brain (**C**) and the liver (**D**) of 3 animals. For each animal, data are presented as the mean of at least 3 images acquired. Statistic with Student's *t*-test: p*< 0.05; 0.005 <p**<0.05; p***<0.001.


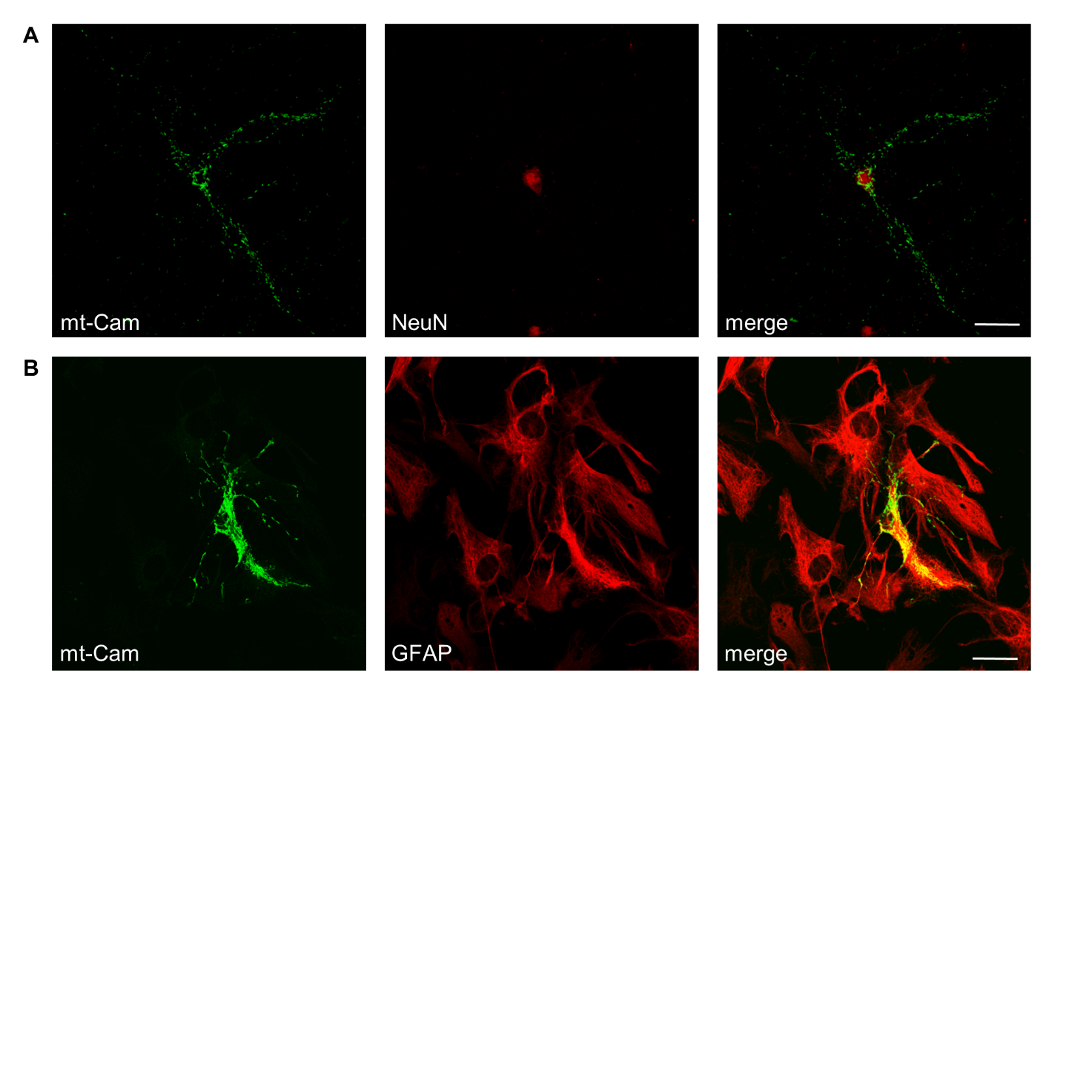


**Supplementary Figure 3. Cultured cortical neurons and astrocytes from mt-Cam-expressing mice. (A-B)** Confocal images of isolated cortical neurons (**A**) or astrocytes (**B**) expressing mt-Cam (green), stained with antibodies against neuronal nuclei marker NeuN (**A**, red) or GFAP (**B**, red). Scale bar 20 µm.


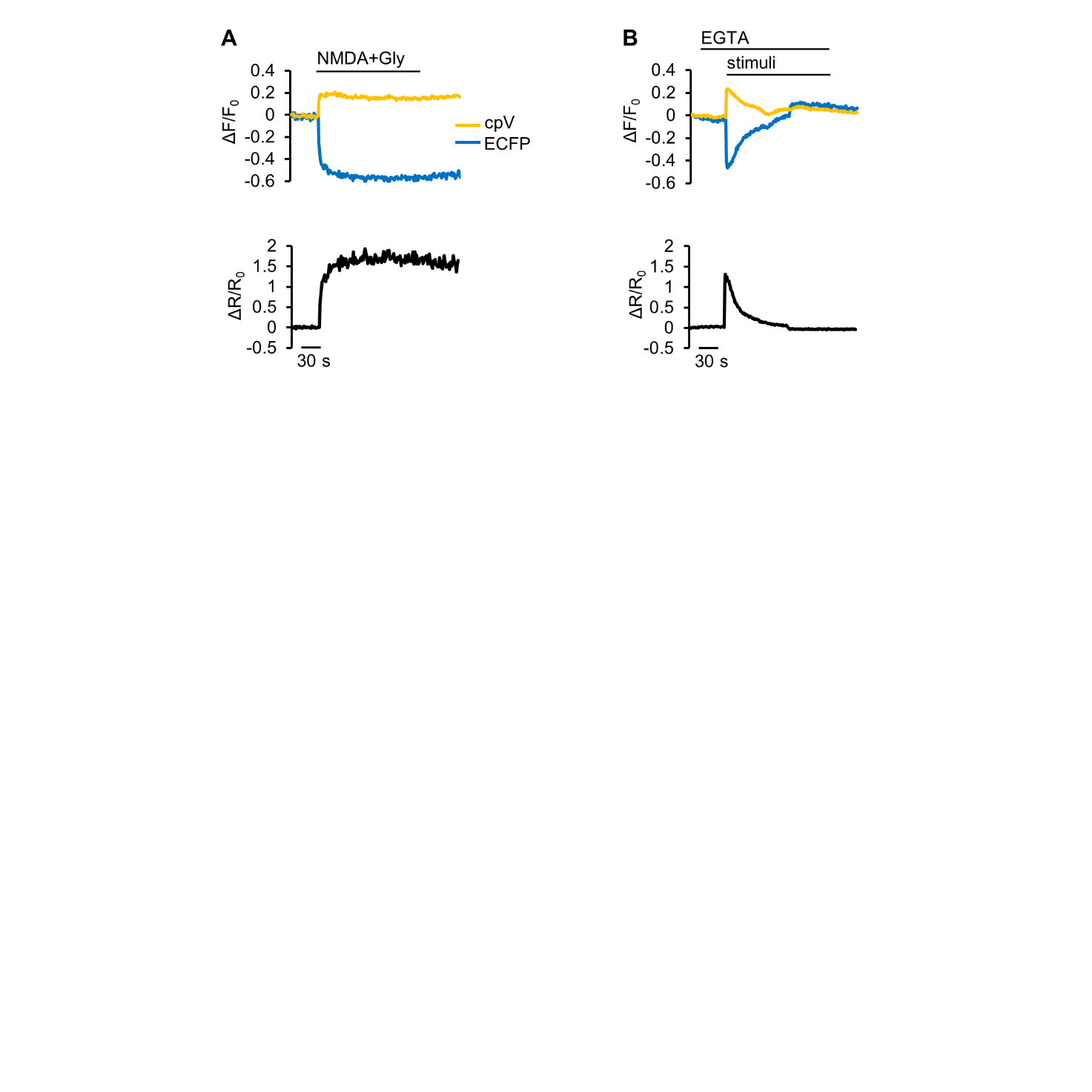


**Supplementary Figure 4. Ca^2+^ imaging in hippocampal neurons expressing mt-Cam**. **(A-B)** Representative traces of hippocampal neurons from mt-Cam-expressing mice upon stimulation with NMDA (20 µM) plus Glycine (40 µM) (**A**) or with a mix of stimuli (DHPG 100 µM, Carbachol 300 µM, ATP 100 µM, Glutamate 100 µM) in the presence of 1 mM EGTA (**B**). Data are presented as ΔF/F_0_ (top), ΔR/R_0_ (bottom).


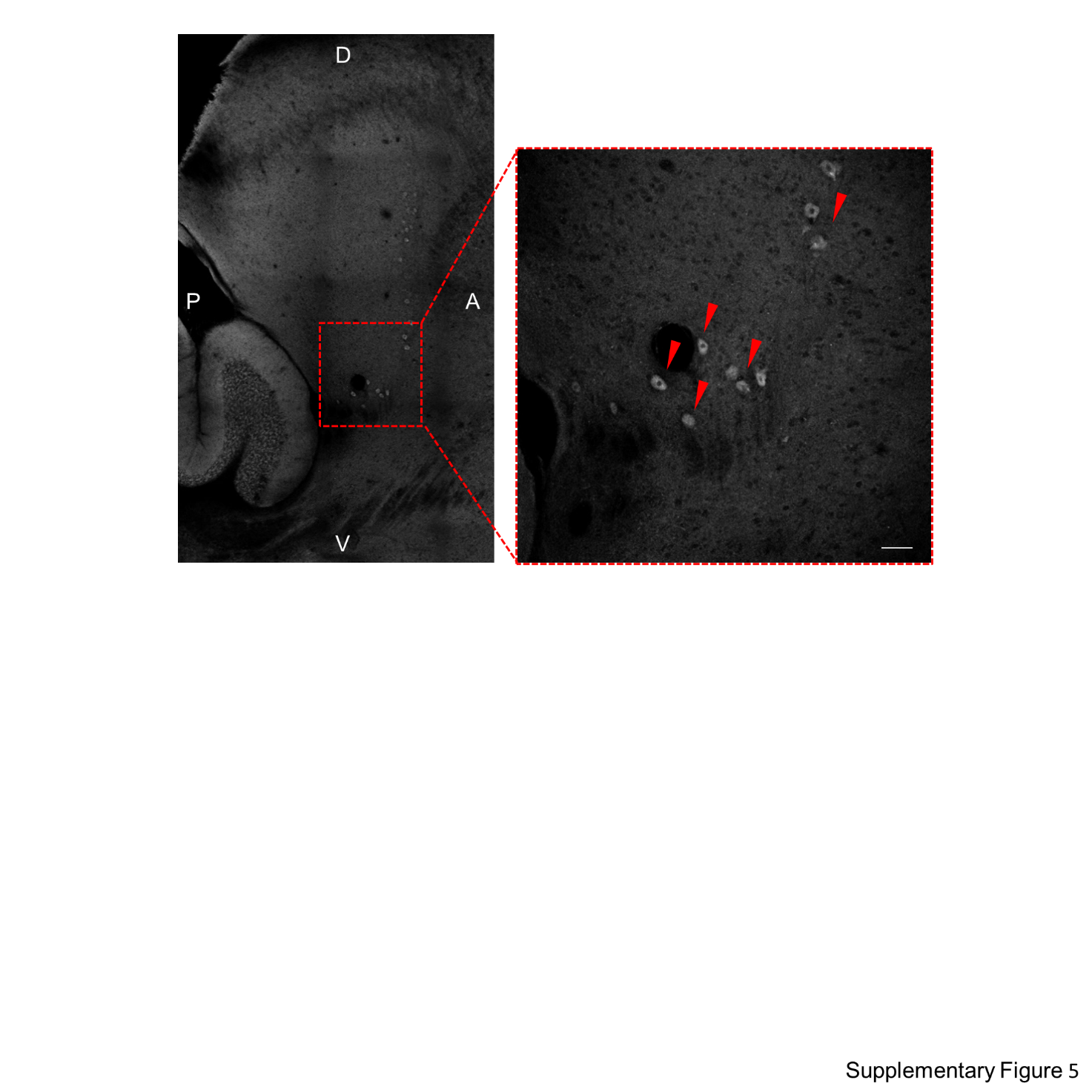


**Supplementary Figure 5**. **mt-Cam expression in cholinergic neurons.** Confocal images of a brain sagittal section of a mouse expressing mt-Cam upon crossing of ROSA26-mt-Cam with ChAT-Cre mice. Positive cholinergic neurons are visible in a small portion of the posterior (P) part of the brain. A = anterior, P = posterior, D = dorsal; V = ventral. Scale bar 50 µm.
